# Mask Wearing Hesitancy During the COVID-19 Pandemic in South Iran

**DOI:** 10.1017/dmp.2021.72

**Published:** 2021-03-10

**Authors:** Ali A. Asadi-Pooya, Abdullah Nezafat, Saeid Sadeghian, Mina Shahisavandi, Seyed Ali Nabavizadeh, Zohreh Barzegar

**Affiliations:** 1 Epilepsy Research Center, Shiraz University of Medical Sciences, Shiraz, Iran; 2 Jefferson Comprehensive Epilepsy Center, Department of Neurology, Thomas Jefferson University, Philadelphia, USA; 3 Department of Pediatric Neurology, Golestan Medical, Educational, and Research Center, Ahvaz Jundishapur University of Medical Sciences, Ahvaz, Iran

**Keywords:** coronavirus, COVID-19, epilepsy, mask, seizure

## Abstract

**Purpose:**

The aim of the current study was to investigate the prevalence of face mask wearing among different groups of people in south Iran. We also investigated the associations between mask wearing hesitancy and various factors.

**Methods:**

We surveyed a sample (convenience sampling) of 5 groups of people: general population, people with epilepsy, people with diabetes mellitus (DM), people with cardiac problems, and people with psychiatric problems. The survey included 4 general questions (age, sex, education, and medical/psychiatric problem) and 4 coronavirus disease 2019 (COVID-19)-specific questions (contracting COVID-19, relatives with COVID-19, wearing a face mask while in crowded places, and the frequency of daily hand washings).

**Results:**

A total of 582 people (153 people with epilepsy, 127 patients with DM, 98 people with cardiac problems, 96 patients with psychiatric disorders, and 108 healthy individuals) participated. Twenty-eight (4.8%) people expressed that they do not wear a face mask when at crowded places. A lower education and less frequent daily hand washings had associations with mask wearing hesitancy.

**Conclusions:**

Mask wearing hesitancy is a concern during a respiratory viral disease pandemic. Paying attention to personal variables, especially if they are modifiable (eg, education and hygiene), is probably productive and practical in promoting mask wearing culture.

Since the onset of the year 2020, the world has been experiencing a catastrophic and fatal pandemic of a coronavirus disease 2019 (COVID-19) caused by severe acute respiratory syndrome coronavirus 2 (SARS-CoV-2).^1^ This virus has a high potential for human to human transmission and may cause a severe illness characterized by acute respiratory distress syndrome, multi-organ failure, and death.^1^ In the absence of an effective treatment against this virus, the options for control are limited to social distancing measures, good hygiene, and wearing a face mask. Coronavirus pathogens are transmitted by droplets and wearing a face mask can reduce the risk of COVID-19 infection.^2,3^ Therefore, wearing a face mask has been widely recommended (or even mandated) to the general public in most countries during the COVID-19 pandemic.^4^

A previous observational study from the United States showed that 77% of all people used face masks in an appropriate way, covering their nose and mouth, while 23% were either incorrectly masked or not masked at all.^5^ The phenomenon of mask wearing hesitancy during this pandemic has also been observed in many other places in the world.^6^ Aside from social and political reasons that may contribute to mask wearing hesitancy (or even anti-mask movements) among the public,^7^ it is important to investigate the prevalence and also the associated risk factors of mask wearing hesitancy in different communities.

The aim of the current study was to investigate the prevalence of face mask wearing hesitancy among people in south Iran. We also investigated the associations between mask wearing hesitancy and various factors (eg, demographic [ie, sex, age, education] and medical [ie, chronic problems]). We surveyed 5 different groups of people (ie, a group of the general population without a history of any chronic medical/psychiatric problems, people with epilepsy, people with diabetes mellitus (DM), people with cardiac problems, and people with psychiatric problems) to investigate the associated factors of mask wearing hesitancy. This information would help public health policy-makers to provide a targeted message to the people on the issue of the necessity and helpfulness of wearing a face mask to help curb this pandemic.

## Methods

In this cross-sectional study, we surveyed a sample (convenience sampling) of 5 groups of people during September, 2020: a group of the general population from Shiraz (a major city in south Iran) without a history of any chronic medical/psychiatric problems (people from the general public in the streets of downtown Shiraz), and 4 groups of patients (based on consecutive referrals) people with epilepsy (referring to the neurology clinic at Shiraz University of Medical Sciences), people with DM (referring to the DM clinic at Shiraz University of Medical Sciences), people with cardiac problems (referring to the cardiology clinic at Shiraz University of Medical Sciences), and people with psychiatric problems (people with depression or anxiety referring to the psychiatry clinic at Shiraz University of Medical Sciences). The inclusion criteria were adults (≥ 18 y) and literacy (> 5 y of education). The exclusion criteria included intellectual disability, psychosis, and unwillingness to participating in the study.

The survey included 4 general questions (age, sex, education [school vs college], and medical/psychiatric problem). It also included 4 COVID-19 specific questions (contracting COVID-19 [self-declared], relatives [ie, spouse, children, siblings, parents] with COVID-19 [self-declared], wearing a face mask while at crowded places, and the frequency of daily hand washings) (Appendix 1).

Statistical analyses were performed using independent t-test, Fisher’s exact test, Pearson chi-squared test, and Bonferroni correction test. Variables with a *P* value < 0.1 in univariate tests were assessed in a logistic regression model. Odds ratio and 95% confidence interval (CI) were calculated. A *P*-value (2-sided) less than 0.05 was considered as significant.

### Standard Protocol Approvals, Registrations, and Patient Consents

The Shiraz University of Medical Sciences Institutional Review Board approved this study (IR.SUMS.REC.1399.1103).

## Results

The total number of the participants was 582 people (153 people with epilepsy, 127 patients with DM, 98 people with cardiac problems, 96 patients with psychiatric disorders, and 108 healthy individuals). The mean age of the participants was 37 y (standard deviation: 15 y) (range: 18 to 97 y). They included 323 females and 259 males. Totally, 28 (4.8%) people expressed that they do not wear a face mask when at crowded places.

Table 1 shows the factors associated with mask wearing hesitancy in this study. Patients with epilepsy had the highest prevalence of mask wearing hesitancy (10%; *P* = 0.0001). In the whole group of the participants, less frequent daily hand washings was associated with mask wearing hesitancy. A lower educational achievement and a younger age showed trends to be associated with mask wearing hesitancy.

**Table 1.** Factors associated with mask wearing hesitancy in univariate analysis

	Not wearing a face mask (*N* = 28)	Wearing a face mask (*N* = 554)	*P*-Value*
Sex (female: male)	12: 16	311: 243	0.17
Mean age ± standard deviation (years)	31 ± 12	38 ± 15	0.01
Education (college)	6 (21%)	224 (40%)	0.04
Frequency of daily hand washings	13 ± 24	31 ± 30	**0.001**
Relatives with COVID-19	8 (29%)	104 (19%)	0.21
History of COVID-19	3 (11%)	47 (8%)	0.72
Chronic disease (none, epilepsy, DM, cardiac, psychiatric)	9: 15: 0: 2: 2	99: 138: 127: 96: 94	**0.0001**

* After Bonferroni correction, a significant predictive value is 0.007. The significant *P* values are in boldface.

We then analyzed the association between mask wearing hesitancy and variables with a *P* < 0.1 in the whole studied group in a binary logistic regression model. The model that was generated by regression analysis was significant (*P* = 0.0001) and could predict the mask wearing hesitancy in 95% of the people. Table 2 shows the results of this analysis. A lower education and less frequent daily hand washings had independent associations with mask wearing hesitancy. A younger age showed a trend and medical/psychiatric problem lost its significance (*P* = 0.12).

**Table 2.** Factors associated with mask wearing hesitancy in a logistic regression model

	Odds ratio	95% confidence interval	*P*-Value
Lower education	4.54	1.66- 12.41	0.003
Less frequent daily hand washings	1.02	1.00- 1.04	0.040
Younger age	1.03	0.99- 1.06	0.063

## Discussion

The use of face masks is part of a comprehensive package of the preventative measures that may limit the spread of certain respiratory viral pathogens, including SARS-CoV-2.^4^ On the other hand, using face masks by people in the community may cause more awareness about the necessity of hand hygiene and the vital role of social distancing during this pandemic.^8^ Face masks may be used either for protection of healthy persons (worn to protect oneself when in contact with an infected person) or for source control (worn by an infected person to prevent onward transmission of the virus).^4^ However, for this preventative strategy to be effective, a widespread public mask wearing compliance is necessary. In the current study, we observed that approximately 5% of the people in south Iran showed mask wearing hesitancy. This rate in a study from the United States was 9%,^5^ which is comparable to our observation. Online surveys in 206,729 people residing in 9 low- and middle-income countries showed that 32.7-99.7% of the respondents used face masks with significant differences across different age groups and genders.^9^

The different prevalence rates of mask wearing hesitancy among various nations could have social, political, and cultural underpinnings, among other variables.^5-7,9^ In countries where wearing face masks is mandatory or highly encouraged (like in Iran) by the government, adherence rates are more than 90%.^9^ Paying attention to the personal variables, especially if they are modifiable, is probably productive and more practical in promoting the culture of mask wearing. In the current study, we observed that lower education and less frequent daily hand washings had associations with mask wearing hesitancy. It seems that people with lower education and those who are not amenable to keeping a good hygiene, are more likely to refuse wearing face masks when it is a necessary practice (ie, at crowded places). Educating the public on the significance of these measures, specifically in a tailored manner (eg, with an understandable language for different people with different levels of education) is an important strategy that should be considered by public health policy-makers.

Of interest, we observed that people with epilepsy and those without any underlying medical problems more often had mask wearing hesitancy than those with underlying medical problems (ie, DM, cardiac problems) (Table 1). This is probably due to the fact that people with underlying medical problems have been identified as high-risk groups, and these people have been advised to practice more precautions.

Considering the transmission route of SARS-CoV-2, wearing face masks is an essential measure to prevent virus transmission, as well as to reduce the hand-to-face contact.^8,10^ Therefore, rational guidance and appropriate educational measures should be provided to the public concerning the use of face masks. The content of messages to the public should generally include: the necessity of wearing the mask, selection of the proper masks, appropriate handling of face masks, and other prevention and control measures that should not be neglected.^10^

Our study has some limitations. The actual representativeness of the participants for the general public is not known, and also it is possible that participants with a positive attitude were more likely to participate in such a survey. Furthermore, the structure and language of the survey might have influenced the results. Future studies should include larger and more diverse sample sizes.

## Data Availability

The data are confidential and will not be shared as per the regulations of Shiraz University of Medical Sciences.
